# N‐acetylglutamate synthase deficiency with associated 3‐methylglutaconic aciduria: A case report

**DOI:** 10.1002/jmd2.12318

**Published:** 2022-07-22

**Authors:** Arthavan Selvanathan, Kalliope Demetriou, Matthew Lynch, Michelle Lipke, Carolyn Bursle, Aoife Elliott, Anita Inwood, Leanne Foyn, Brett McWhinney, David Coman, Jim McGill

**Affiliations:** ^1^ Queensland Lifespan Metabolic Medicine Service Queensland Children's Hospital Brisbane Australia; ^2^ Chemical Pathology, Central Laboratory Pathology Queensland Herston Australia; ^3^ School of Medicine University of Queensland Brisbane Australia

**Keywords:** 3‐methylglutaconic aciduria, mitochondrial dysfunction, N‐acetylglutamate synthase deficiency, Urea cycle disorder

## Abstract

N‐acetylglutamate synthase (NAGS) deficiency is a rare autosomal recessive disorder, which results in the inability to activate the key urea cycle enzyme, carbamoylphosphate synthetase 1 (CPS1). Patients often suffer life‐threatening episodes of hyperammonaemia, both in the neonatal period and also at subsequent times of catabolic stress. Because NAGS generates the cofactor for CPS1, these two disorders are difficult to distinguish biochemically. However, there have now been numerous case reports of 3‐methylglutaconic aciduria (3‐MGA), a marker seen in mitochondrial disorders, occurring in CPS1 deficiency. Previously, this had not been reported in NAGS deficiency. We report a four‐day‐old neonate who was noted to have 3‐MGA at the time of significant hyperammonaemia and lactic acidosis. Low plasma citrulline and borderline orotic aciduria were additional findings that suggested a proximal urea cycle disorder. Subsequent molecular testing identified bi‐allelic pathogenic variants in *NAGS*. The 3‐MGA was present at the time of persistent lactic acidosis, but improved with normalization of serum lactate, suggesting that it may reflect secondary mitochondrial dysfunction. NAGS deficiency should therefore also be considered in patients with hyperammonaemia and 3‐MGA. Studies in larger numbers of patients are required to determine whether it could be a biomarker for severe decompensations.

**Synopsis**

We present the first published case of a patient with N‐acetylglutamate synthase (NAGS) deficiency who had 3‐methylglutaconic aciduria at the time of presentation. Previous reports suggested that the presence of 3‐methylglutaconic acid helped distinguish CPS1 from NAGS.

## 1 INTRODUCTION

N‐acetylglutamate synthase (NAGS) deficiency (OMIM #237310) is a rare urea cycle disorder, with only approximately 100 cases reported in the literature to date. The defect in the NAGS enzyme (EC 2.3.1.1) prevents production of the co‐factor NAG, which is required for optimal activity of a rate‐limiting enzyme of ureagenesis, carbamoylphosphate synthetase 1 (CPS1, EC 6.3.4.16). Deficiency of NAGS therefore results in hyperammonaemia, which can present in the neonatal period and be life‐threatening.^1^ Distinguishing between NAGS deficiency and CPS1 deficiency (OMIM #237300) is difficult from a biochemical perspective, as both have hyperammonaemia, low citrulline, and normal orotate: hence, molecular testing is often required. NAGS deficiency can be amenable to treatment with carglumic acid, and so the distinction also carries therapeutic significance.^2^

Previously 3‐methylglutaconic aciduria (3‐MGA) had been reported in many cases of neonatal‐onset CPS1 deficiency,^3^, ^4^ as well as patients with ornithine transcarbamylase (OTC) deficiency and argininosuccinate lyase (ASL) deficiency,^5^ but not in NAGS deficiency.^4^ The 3‐MGA is often a marker of mitochondrial dysfunction, either primary or secondary,^6^ and occurred in these cases with concomitant elevations in serum lactate.

We report the first patient in the literature with NAGS deficiency and 3‐MGA at the time of diagnosis. This patient presented with severe neonatal‐onset hyperammonaemia, with associated persistent elevation in lactate, suggesting that the 3‐MGA is more indicative of secondary mitochondrial dysfunction, rather than a disease‐specific finding. Further study of other patients with confirmed NAGS deficiency is required to establish whether 3‐MGA may be useful as a biomarker of severe metabolic decompensation.

## 2 CASE REPORT

A four‐day‐old male infant was brought in by ambulance to the local hospital because of respiratory distress. This was the third child to a nonconsanguineous couple, with no family history of any genetic disorders.

The baby had been born at 41 weeks gestation weighing 2.95 kg, with APGAR scores of 9 (at one and five minutes of age) and was discharged home within a day. Standard infant formula feeds had been commenced from birth, with no concerns in the first 24 hours of life. Subsequently the baby developed progressive lethargy with poor feeding and had not woken for feeds for over 12 hours at the time of presentation.

On examination, the infant was poorly responsive. He had delayed central capillary refill of five seconds, with only partial improvement with two 20 ml/kg boluses of 0.9% NaCl. There was marked respiratory distress and tachypnoea, with posturing and hypertonicity of the upper limbs also noted. Pupils were equal (3 mm) and reactive to light, and the fontanelle was not bulging. Femoral pulses were difficult to palpate. Abdominal and cardiorespiratory examinations were otherwise normal.

The initial blood gas demonstrated a metabolic acidosis without respiratory compensation (pH 7.24, bicarbonate 18 mmol/L, base excess −7.7 mmol/L, pCO2 42 mmHg), with blood glucose of 5.8 mmol/L (reference range [RR], 3.9–5.8 mmol/L) and significantly elevated lactate of 8.7 mmol/L (RR 0.5–2.0 mmol/L). The baby was intubated and ventilated, commenced on antibiotics and transferred to the nearest tertiary pediatric center. A prostaglandin infusion was also commenced, as well as intravenous fluids with 10% dextrose at maintenance rate; the former was ceased once an echocardiogram had ruled out a duct‐dependent cardiac lesion.

A serum ammonium level was markedly elevated at 1712 umol/L (RR <100 umol/L). Intravenous benzoate and arginine loading infusions were commenced, as well as a carnitine infusion, shortly after which haemofiltration was initiated. Despite this, the ammonium level was 500 umol/L after 24 hours and only normalized after 48 hours.

Plasma amino acid analysis showed a marked elevation in glutamine of 2700 umol/L (RR <960 umol/L) and alanine of 1800 umol/L (RR <410 umol/L), in keeping with the hyperammonaemia and lactic acidosis respectively. Citrulline was significantly decreased, (1 umol/L, RR 10‐30 umol/L) and ornithine elevated (140 umol/L, RR <110 umol/L), suggesting a proximal urea cycle disorder.

Urine organic acid analysis revealed marginal elevation in orotate (3.7 mmol/mol creatinine, RR <2.2 mmol/mol creatinine) which was considered unlikely to be significant diagnostically, as well as increased 3‐methylglutaconic acid (61 mmol/mol creatinine, RR <12 mmol/mol creatinine) and 3‐methylglutaric acid (17 mmol/mol creatinine, RR <4.6 mmol/mol creatinine). Fumarate (58 mmol/mol creatinine, RR <26 mmol/mol creatinine) and malate (157 mmol/L, RR <85 mmol/mol creatinine) were also mildly elevated. The 3‐MGA improved to 12 mmol/mol creatinine once the serum lactate had normalized. Brain magnetic resonance imaging demonstrated both acute changes (T2 hyperintensity in the posterior limb of the internal capsule) and evidence of right middle cerebral artery territory infarction. Given the presence of 3‐MGA, a provisional diagnosis of CPS1 deficiency was made, in keeping with the revised urea cycle disorder guidelines.^4^

Next generation sequencing of genes associated with urea cycle disorders identified bi‐allelic pathogenic variants in *NAGS* (c.622C > T|p.Arg208* and c.1368_1369delinsT|p.Gly457Alafs*110), consistent with NAGS deficiency. The patient had sustained a substantial neurological insult during this time, resulting in persistent irritability, hypertonia, and seizures requiring multiple antiepileptics. The molecular diagnosis was identified one month after the initial neurological insult: hence, carglumic acid was not pursued as a treatment option in view of his severe acquired brain injury and subsequent refractory status epilepticus.

## 3 DISCUSSION

### 3.1 Pathophysiology of NAGS deficiency

The urea cycle is the main pathway in humans for conversion of nitrogenous waste products into urea, which can be excreted in the urine. Defects in any of the enzymes or transporters in this pathway results in build‐up of ammonia and associated clinical features.

The first rate‐limiting step in the pathway is CPS1, which combines bicarbonate, ammonia, and adenosine triphosphate to generate carbamoylphosphate. The cofactor, NAG, is crucial to CPS1 function and is synthesized by the NAGS enzyme from acetyl‐Coenzyme A and glutamate. As a result, deficiency in NAGS reduces the availability of NAG for use by CPS1, effectively limiting the ability of this enzyme to function and causing hyperammonaemia.^1^

NAGS deficiency is the rarest of the urea cycle disorders, with only approximately 100 cases reported to‐date.^2^ The majority of diagnosed patients have presented with neonatal‐onset disease, manifesting with poor feeding, vomiting, lethargy, tone abnormalities, tachypnoea and seizures.^7^ Similar to the other urea cycle disorders, late‐onset forms have also been reported.^8^, ^9^ Management of the condition involves dietary protein restriction, ammonia scavenging agents and carglumic acid (the latter generally dosed at 10‐100 mg/kg/day, increasing to 250 mg/kg/day with acute hyperammonaemia).^4^ In most cases, carglumic acid is not able to be used as monotherapy.^2^

The biochemical picture of NAGS deficiency (low citrulline, high glutamine and ammonia, normal orotate excretion) is identical to CPS1 deficiency. Liver enzyme activity measurements, molecular testing, or a trial of carglumic acid offer ways of distinguishing between the two conditions. However, CPS1 liver enzyme activity can be low in patients with NAGS deficiency, hence molecular testing is often preferred for diagnosis.^10^ 3‐MGA has been reported in CPS1 deficiency,^3^, ^4^, ^11^ but has not previously been seen in NAGS deficiency.

### 3.2 3‐MGA and its association with human disease

3‐MGA occurs in a variety of conditions^6^ (see Table 1). 3‐methylglutaconic acid is an intermediate of leucine catabolism and a defect in this pathway (of 3‐methylglutaconyl‐CoA hydratase, EC 4.2.1.18) is responsible for 3‐MGA Type 1.^12^ In this condition, there is associated elevation in 3‐hydroxyisovalerate (3‐HIVA) and 3‐methylglutrate (3‐MG) in keeping with distal blockade. However, for the remaining conditions, 3‐MGA is thought to be a secondary phenomenon occurring independently of this pathway. Leucine loading does not appear to increase 3‐MGA excretion in these other conditions^13^ and 3‐HIVA excretion is normal.^14^

**TABLE 1:** Causes of 3‐methylglutaconic aciduria ‐ molecular basis and key clinical features

Cause of 3‐MGA	Gene	Clinical features
Type I	*AUH*	Likely asymptomatic in childhood, late‐onset leukoencephalopathy
Type II (Barth Syndrome)	*TAZ*	Short stature, cardiomyopathy, myopathy, neutropenia, and hypocholesterolaemia (X‐linked)
Type III (Costeff Syndrome)	*OPA3*	Optic atrophy, movement disorder, and spastic paraplegia
Type IV	—	Heterogenous group of disorders including *TMEM70* deficiency
Type V	*DNAJC19*	Cardiomyopathy, cardiac conduction defects, cerebellar ataxia, testicular dysgenesis, and failure to thrive
Type VI (MEGDEL Syndrome)	*SERAC1*	Hypoglycaemia, liver failure, encephalopathy, deafness, Leigh‐like syndrome, and hyperammonaemia
Type VII	*CLPB*	Cataracts, neurological disease, and neutropenia
Type VIII	*HTRA2*	Early‐onset epileptic encephalopathy, hypotonia, abnormal movements, and apnoeas
Type IX	*TIMM50*	Early‐onset epileptic encephalopathy, hypotonia, and spasticity
Other causes of 3‐MGA	—	Small molecule disorders (including urea cycle disorders)
		Other primary mitochondrial disorders
		Nonmetabolic disorders (hematological, neuromuscular, other genetic conditions)
		Pregnancy

This “secondary” 3‐MGA is thought to occur due to nonspecific mitochondrial dysfunction. Su and Ryan (2014) suggest that altered mitochondrial metabolism results in increased NADH in the inner mitochondrial space, inhibiting citric acid cycle enzymes and causing accumulation of acetyl‐CoA.^15^ The enzymes at the end of the leucine catabolic pathway catalyze reversible reactions; hence this excess acetyl‐CoA can be converted to acetoacetyl‐CoA (by beta‐ketothiolase, EC 2.3.1.16), which in turn is converted to 3‐hydroxymethylglutaryl‐CoA (by HMG‐CoA synthase, EC 2.3.3.10), and then back to 3‐methylglutaconyl‐CoA. 3‐methylcrotonyl‐CoA carboxylase is an essentially irreversible reaction, leading to accumulation of 3‐methylglutaconyl‐CoA, and excretion of 3‐methylglutaconic acid in the urine.

As well as occurring in the primary and secondary 3‐MGAs, 3‐MGA can also occur in other inborn errors of metabolism. It is seen most commonly with deficiency of the enzyme hydroxymethylglutaryl‐CoA (HMG‐CoA) lyase, the next step in the leucine catabolic pathway following 3‐methylglutaconyl‐CoA hydratase.^16^ However, it has also been seen in other small molecule disorders that do not directly impair leucine catabolism. Wortmann et al. (2013) reported 227 patients with 3‐MGA,^5^ with the most common aetiologies being other small molecule disorders (61 patients), primary mitochondrial disorders (49 patients) and nonmetabolic disorders (including genetic and neuromuscular conditions; 43 patients). Six of the 61 patients with small molecule disorders had a urea cycle disorder (CPS1, OTC, and ASL deficiencies). The reasons for secondary mitochondrial dysfunction in these disorders are not entirely clear; however direct inhibition of alpha‐ketoglutarate dehydrogenase by high levels of ammonia has been demonstrated in rodent models.^17^

In a similar vein, Rokicki et al. (2017) noted 3‐MGA at the time of diagnosis, in nine of ten neonates with CPS1 deficiency.^3^ Most of these patients also had lactate elevation. There are also other known conditions that can cause the combination of hyperammonaemia, lactic acidosis and 3‐MGA, specifically *SERAC1* deficiency (OMIM #614739) or *TMEM70* deficiency (OMIM #614052).^18^, ^19^ Clarification of the diagnosis on clinical, biochemical, and molecular grounds is crucial in order to ensure tailored treatment.

In the current case, a diagnosis of CPS1 deficiency was initially considered, as suggested by the diagnostic algorithm in the most recent urea cycle disorder guidelines.^4^ However, the molecular results subsequently confirmed a diagnosis of NAGS deficiency. As such, this condition should also be considered within the differential diagnosis for patients with 3‐MGA where a proximal urea cycle disorder is suspected, and a trial of carglumic acid initiated if the treatment focus is still active management. In some centers, the trial of carglumic acid is given as part of the initial management, and this could be considered as an empirical treatment to account for the rarer possibility of NAGS deficiency. A response to carglumic acid is also diagnostically useful in distinguishing NAGS and CPS1 deficiencies.

## 4 CONCLUSION

We present the first reported case of 3‐MGA in NAGS deficiency. Whilst the elevated lactate may be non‐specific in a critically unwell infant, the additional findings of increased malate and fumarate in our patient are consistent with previous literature suggesting that 3‐MGA is a secondary finding, which occurs due to mitochondrial dysfunction in a range of inborn errors of metabolism. Whilst we were not able to serially monitor the extent of the 3‐MGA in our patient over time, it may be an indicator of severity of metabolic decompensation. This may be useful in patients with other urea cycle disorders with more variable phenotypes such as OTC deficiency. NAGS deficiency should be considered within the differential diagnosis for patients with 3‐MGA where a proximal urea cycle disorder is suspected; a trial of carglumic acid should be initiated if active management is being pursued.

## AUTHOR CONTRIBUTIONS

Arthavan Selvanathan: conception and design, ethics submission, collecting data, writing manuscript, reviewing manuscript.

Kalliope Demetriou: analysis/interpretation of data, drafting of article.

Matthew Lynch: analysis/interpretation of data, drafting of article.

Michelle Lipke: analysis/interpretation of data, drafting of article.

Carolyn Bursle: analysis/interpretation of data, drafting of article.

Aoife Elliott: analysis/interpretation of data, drafting of article.

Anita Inwood: conception and design, ethics submission, analysis/interpretation of data, drafting of article, reviewing manuscript.

Leeanne Foyn: analysis/interpretation of data, drafting of article.

Brett McWhinney: analysis/interpretation of data, drafting of article.

David Coman: conception and design, analysis/interpretation of data, drafting of article, reviewing manuscript.

Jim McGill: conception and design, analysis/interpretation of data, drafting of article, reviewing manuscript.

## CONFLICT OF INTEREST

We declare no conflicts of interest.

## ETHICS STATEMENT

Approval for publication was obtained from the local Human Research Ethics Committee (Case Report #1338344).

## FUNDING INFORMATION

There were no sources of funding that were required for this project.

## Data Availability

Data supporting the results reported in this article are not publicly available and are stored within the hospital and pathology electronic medical records. Further information regarding these results could be made available on request.
